# The truth in complexes: perspectives on ion channel signaling nexuses in the nervous system

**DOI:** 10.3389/fncel.2014.00406

**Published:** 2014-11-26

**Authors:** Leigh A. Swayne, Christophe Altier, Gerald W. Zamponi

**Affiliations:** ^1^Island Medical Program, Division of Medical Sciences, University of VictoriaVictoria, BC, Canada; ^2^Department of Biology, University of VictoriaVictoria, BC, Canada; ^3^Department of Cellular and Physiological Sciences, University of British ColumbiaVancouver, BC, Canada; ^4^Department of Physiology and Pharmacology, University of CalgaryCalgary, AB, Canada

**Keywords:** interactome, ion channels, signaling networks, protein-protein interactions, protein-lipid interactions

## Ion channels as signaling nexuses

Ion channels are complex hetero-oligomeric structures characterized by large, dynamic interaction networks, or “interactomes.” In addition to directing channel localization, density and ion fluxes, these complexes facilitate downstream signaling events. Moreover, pathological modulation of these networks contributes to neurological dysfunction. Our contributors to this Research Topic, *“The truth in complexes: why unraveling ion channel multi-protein signaling nexuses is critical for understanding the function of the nervous system”* have considered interactomes from the perspective of the ion channel, from that of its intracellular protein modulators, and even from the point of view of lipid modulators. Together these diverse perspectives spin an intricate web of ion channel regulation in the nervous system.

## Major hub: the N-methyl-D-Aspartate Receptor (NMDAR)

Described by Fan et al. (2014) as a “multifunctional machine,” the NMDAR interacts with a staggering number of proteins to shape synaptic plasticity, psychiatric disorders and ischemic neuronal damage. Notably, the authors outline arguably the most exciting example of interactome-based basic science leading to improved health outcomes: Tat-NR2B9c (also called NA-1). This cell-permeable peptide targets a specific NMDAR interaction, reducing ischemic brain damage in rodents, primates and humans (Sun et al., 2008; Cook et al., 2012; Hill et al., 2012). Li et al. (2014) similarly highlights interactions between several ligand-gated channels, including the NMDAR with other receptors and intracellular proteins, again focusing on these interactions as potential therapeutic targets for neuroprotection.

## Novel nodes

Several other contributions shed light on the new insights into the function and composition of interactomes of various voltage-gated channels, regulated leak channels, and so called large pore channels.

### Voltage-gated channels

Traditionally viewed as auxiliary subunits, K^+^ channel regulatory proteins are growing in complexity in terms of function and type. Known to regulate activation and trafficking of muscarinic receptor-activated Kir3 channels, Zylbergold et al. (2014) provide evidence for an additional role of Gβγ subunits in Kir3 channel stability. Nagi and Pineyro (2014) focus specifically on opioid receptor signaling in the regulation of these channels. Jerng and Pfaffinger (2014) describe regulation of another K^+^ current, sub-threshold A-type (Kv4), by the so-called auxiliary subunits, dipeptidyl peptidase-like proteins (DPLPs) and Kv4 channel interacting proteins (KChIPs). While these were amongst the first identified interactors (e.g., for KChiP An et al., 2000), subsequent studies have significantly expanded the network. With respect to DPLPs and KChIPs, further study has also shed new light on their molecular diversity via alternative splicing as well as their roles in regulating several other channel types, such as voltage-gated Ca^2+^ channels and NMDARs. Connecting K^+^ channels with voltage-gated Ca^2+^ channels, Engbers et al. (2013) review how channel-channel interactions between intermediate conductance Ca^2+^-activated K^+^ channels (IKCa) and low voltage-activated Ca^2+^ channels (Cav3) functionally interact with other conductances to regulate signal processing in the cerebellum.

### Na^+^ leak channel, NALCN

Elusive until recently, understanding of this regulated leak channel whose loss in mice is lethal (Lu et al., 2007), has greatly expanded by virtue of key insights into its interactome. Cochet-Bissuel et al. (2014) detail its ever-expanding list of interacting proteins, such as the M3 muscarinic receptor (Swayne et al., 2009). The authors highlight the involvement of the NALCN interactome in a number of disorders in the nervous system ranging from autism spectrum disorder (ASD) and schizophrenia to epilepsy and Alzheimer's disease.

### Pannexin 1 (Panx1)

Permeable to ions and small metabolites like ATP, Panx1 channels gained early notoriety as “death pores” in ischemic stroke and seizure (Thompson et al., 2006, 2008; Weilinger et al., 2012). Highly expressed in neonatal brain (Ray et al., 2005; Vogt et al., 2005), Panx1 also positively regulates proliferation and differentiation, and negatively regulates neurite outgrowth in developing neurons (Wicki-Stordeur et al., 2012; Wicki-Stordeur and Swayne, 2013). Wicki-Stordeur and Swayne (2014) reviewed the growing Panx1 interactome to shed clues on the signaling pathways in which Panx1 might be involved, highlighting roles in cytoskeletal remodeling and inflammation.

## Multi-tasking intracellular modulators

A number of contributions underscore the capacity of “promiscuous” intracellular proteins to modulate a variety of ion channels and receptors through physical interaction. Reviewed by Donnelier and Braun (2014), cysteine string protein (CSP) is a resident pre-synaptic vesicle molecular chaperone targeting ion channels and vesicle-trafficking proteins. Not surprisingly, loss of, or mutation in CSP leads to synaptic dysfunction and neurodegeneration in a variety of systems (e.g., Zinsmaier et al., 1994; Fernandez-Chacon et al., 2004; Noskova et al., 2011). The sigma-1 receptor, reviewed by Pabba (2013), is an intracellular transmembrane protein that also acts in a chaperone-like way, modulating plasma membrane localized voltage- and ligand-gated channels with diverse neurophysiological and neuropathological implications. Harraz and Altier (2014) further link intracellular proteins to the regulation of plasma membrane channels, reviewing Stromal Interaction Molecule 1 (STIM1) in store-operated Ca^2+^ entry. They describe foundational work implicating STIM1 as the Ca^2+^ sensor in this process critical for maintaining neurotransmission. Further they outline key physical interactions between STIM1 with Ca^2+^-release activated channels and voltage-gated Ca^2+^ channels that coordinate the activation and inhibition of these types of channels, respectively. Finally, two papers by Wilson et al. (2014a,b) focus on another intracellular multi-functional/multi-interactome protein, collapsin response mediator protein 2 (Crmp2). Best known as a microtubule stabilizer, Crmp2 is regulated in a context specific way by multiple kinases, and in turn, positively regulates both ligand- and voltage-gated Ca^2+^ channels.

## New frontiers: toward more comprehensive macromolecular networks

Adding further complexity to ion channel networks is consideration of lipid membrane composition and lipid second messengers. In the sole lipidome-oriented contribution, Raboune et al. (2014) identify novel N-acyl amides regulating transient receptor potential vanilloid (TRPV) channels in the context of inflammatory pain. The future understanding of ion channel interactomes will undoubtedly include both proteome and lipidome components as technological advances in lipidomic research (Bou Khalil et al., 2010) become mainstream.

## Final perspectives: interactomes to bedside

While daunting, elucidating these macromolecular intricacies has a translational silver lining: while difficult to identify and unravel, the myriad interaction loci revealed by studying these interactions present unique opportunities for discrete, and potentially safer therapeutic intervention. For example, selective blockade at key interaction loci with cell-permeable peptides now provides an infinite number of ways in which interactomes can be discretely modulated to improve health outcomes.

### Conflict of interest statement

The authors declare that the research was conducted in the absence of any commercial or financial relationships that could be construed as a potential conflict of interest.
